# Effects of Native or Exogenous Benzoxazinoids on In Vitro Ruminal Fermentation and Degradability of Either Fresh or Ensiled Whole‐Crop Maize‐Based Diets in Cattle

**DOI:** 10.1111/jpn.70006

**Published:** 2025-08-22

**Authors:** Claudia Lang, Mubarik Mahmood, Ratchaneewan Khiaosa‐ard, Anna Kaltenegger, Elke Humer, Stefanie Wetzels, Josef J. Gross, Christelle A. M. Robert, Pierre Mateo, Matthias Erb, Qendrim Zebeli, Thomas Hartinger

**Affiliations:** ^1^ Center for Animal Nutrition and Welfare University of Veterinary Medicine Vienna Austria; ^2^ Center for Food Science and Veterinary Public Health University of Veterinary Medicine Vienna Austria; ^3^ Veterinary Physiology, Vetsuisse Faculty University of Bern Bern Switzerland; ^4^ Institute of Plant Sciences, Faculty of Science University of Bern Bern Switzerland; ^5^ Insitute of Animal Science University of Bonn Bonn Germany

**Keywords:** cattle, health risk, Rusitec, secondary plant compound, toxicology

## Abstract

Benzoxazinoids (BXs) are secondary plant compounds and an important part of the plant's defence system against herbivore attacks and microbial pathogens in maize. Whole‐crop maize represents a common feed for ruminant livestock and is most commonly fed as silage, a conservation process that promotes the conversion of BXs into the potent downstream metabolite 6‐Methoxy‐benzoxazolin‐2(3*H*)‐one (MBOA). Possibly, the antimicrobial impact of BXs may not be restricted to plant pathogens but could as well affect the rumen ecosystem, which, however, has not yet been investigated. The present study analysed the effects of a low and a high BX maize genotype, either in fresh or ensiled form, on the in vitro ruminal fermentation and the degradability of main proximate nutrients using a rumen simulation technique. Moreover, the addition of exogenous MBOA when incubating the low BX maize genotype was tested. The data obtained showed no differences in ruminal fermentation variables, such as pH, short‐chain fatty acids, gas production and gas composition. Likewise, the incubation of high BX maize genotype or the addition of exogenous MBOA did not affect the substrate degradability. The ensiling of maize slightly increased the degradability of certain proximate nutrients when compared to fresh maize, which, however, should not be related to maize genotype or exogenous MBOA. No detrimental impact of BXs on the in vitro ruminal fermentation were found and based on this, future studies may then focus on potential absorption and subsequent impact of BXs or its derivatives on the animal in long‐term.

## 1 Introduction

Secondary plant compounds have diverse functions in plants and can exert both beneficial as well as detrimental impacts on ruminants. Prominent representatives for secondary plant compounds are essential oils, saponins and tannins that are comparably well investigated regarding their effects on ruminant digestion and health. For instance, they can improve ruminal nitrogen utilization and lower enteric methane production, but on the other hand, as well reduce voluntary feed intake and cause maldigestion in ruminants (Makkar et al. 1995; Patra and Yu 2012). A yet marginally explored group of secondary plant compounds are benzoxazinoids (BXs), which are part of the plant's defence system against herbivore attacks in different grasses (Poaceae), including maize that is a common feedstuff for livestock (Robert and Mateo 2022). BXs possess a wide range of potent insecticidal as well as antimicrobial effects (Wouters et al. 2016; Robert and Mateo 2022), which is particularly true for its downstream metabolite 6‐methoxy‐2‐benzoxazolinone (MBOA) that was already shown to be effective against different pests, such as the European corn borer (*Ostrinia nubilalis*; Campos et al. 1988). Indeed, MBOA may be of central importance as it constitutes a stable intermediate BX metabolite that is present over longer periods in soil and plants (Macías et al. 2004; Fomsgaard et al. 2004; Gross, Mateo, Ramhold, et al. 2023). However, the impact of MBOA or BXs in general present in maize may not be restricted to insects or plant pathogens (Schlaeppi et al. 2021), but could also negatively affect livestock such as ruminants that typically ingest large amounts of such forages, and therefore are exposed to substantial amounts of BXs (Gross, Mateo, Ramhold, et al. 2023; Gross, Mateo, Schlaeppi, et al. 2023). Yet, data on livestock animals is lacking, but findings from Dai et al. (2022) show compromised growth and a shifted caecal microbiome structure in Brandt's voles (*Lasiopodomys brandtii*) in response to 100 µg MBOA per day.

Indeed, because of the high yield and energy density, whole‐crop maize—predominantly conserved as maize silage—has advanced to become an important forage in the feeding of cattle worldwide (Adesogan et al. 2020). Notably, unlike other secondary plant compounds such as saponins that are degraded in the silo (Kalač et al. 1996), recent research revealed that the ensiling process actually promotes the conversion of BXs into MBOA in maize (Gross, Mateo, Schlaeppi, et al. 2023), indicating a stronger burden for cattle compared to feeding fresh maize. Besides the impact of feed conservation, large variation in BX concentrations among different maize genotypes (Klun and Brindley 1966; Miller and Zhao 2015) may further vary the level to which ruminants are exposed to this group of secondary plant compounds.

A stable rumen fermentation is pivotal for supplying the host with energy and nutrients; thus being essential for both productivity and health. In addition, the rumen is actually the first place of activity of BXs and their interaction with the rumen microbiota. In fact, via a potential carry‐over in animal‐derived foods, BXs—or more likely their emerging downstream metabolites—could even have implications on human health (Adhikari et al. 2015). Thus, in consideration of the one health concept, it is important to assess BX role in the rumen regarding their potential impact on the fermentation, which is yet lacking. In this context, the rumen simulation technique (Rusitec) system (Czerkawski and Breckenridge 1977) has advanced to become a standard tool to test the effects of such substances on rumen fermentation under controlled and standardized conditions (e.g. Seeling et al. 2006; Wetzels et al. 2018; Mahmood et al. 2020).

Consequently, the present study aimed to analyse the effects of BXs and the derivative MBOA on the in vitro rumen fermentation using the Rusitec system. For this, two maize genotypes differing in native BX concentration, i.e., low or high, were incubated as whole‐crop maize in either fresh or ensiled form. Additionally, the low BX genotype was further tested without or with the addition of exogenous MBOA to better ensure that potential differences found in in vitro rumen fermentation are associated with BXs. It was hypothesized that BXs, including exogenous MBOA, negatively affect the ruminal fermentation and compared to the incubation of low BX maize, high BX maize results in a lower yield of short‐chain fatty acids (SCFA) due to an impaired microbial activity, especially when incubating maize in ensiled form.

## 2 Materials and Methods

### 2.1 Biological Resources

Maize seeds (*Zea mays* L.) genotypes of the BX‐producing line W22 (wild‐type) and the BXs mutant *bx1::W22* (referred to as bx1; Tzin et al. 2015) were used. The bx1 line produces residual levels of BXs (< 10%), i.e., this genotype is low in native BX (around 90% less than the wild‐type genotype). Original seeds were kindly provided by Georg Jander (Boyce Thompson Institute, Ithaca, NY, USA) and bred on a large scale in Changings and Posieux, Switzerland, for field assays as described in detail in Gross, Mateo, Schlaeppi, et al. (2023). Consequently, six different diets with whole‐crop maize as the main component were prepared: (i) fresh wild‐type maize [Fresh wild‐type], (ii) fresh bx1 mutant maize [Fresh bx1], (iii) fresh bx1 mutant maize plus 250 µg of exogenous MBOA [Fresh bx1+MBOA], (iv) Ensiled wild‐type maize [Silage wild‐type], (v) ensiled bx1 mutant maize [Silage bx1] and (vi) ensiled bx1 mutant maize plus 250 µg of exogenous MBOA [Silage bx1+MBOA]. The complete diet components and chemical composition of all treatments are provided in Table 1. The exogenous MBOA was purchased from Sigma‐Aldrich, Buchs, Switzerland.

**Table 1:** Overview on diet components and chemical composition of diets (as % DM if not stated otherwise).

		Treatments^a^					
		Fresh			Silage		
	Adaptation diet	Wild‐type	bx1	bx1+MBOA	Wild‐type	bx1	bx1+MBOA
Ingredients							
Fresh whole‐crop maize	0.0	50.0	50.0	50.0	0.0	0.0	0.0
Ensiled whole‐crop maize	50.0	0.0	0.0	0.0	50.0	50.0	50.0
Hay	10.0	10.0	10.0	10.0	10.0	10.0	10.0
Wheat	23.3	23.3	23.3	23.3	23.3	23.3	23.3
Rapeseed meal	15.0	15.0	15.0	15.0	15.0	15.0	15.0
Mineral premix^b^	1.7	1.7	1.7	1.7	1.7	1.7	1.7
Chemical composition							
Dry matter (%)	63.0	60.4	59.5	59.5	59.2	59.2	59.2
Ash	6.5	5.6	5.7	5.7	5.9	5.9	5.9
Organic matter^c^	93.5	94.4	94.3	94.3	94.1	94.1	94.1
Crude protein	16.8	16.4	16.3	16.3	16.8	16.8	16.8
Ether extract	2.1	1.7	1.5	1.5	2.5	1.6	1.6
Neutral detergent fibre	39.2	36.4	38.7	38.7	35.6	37.4	37.4
Non‐fibre carbohydrates^d^	37.4	39.9	37.8	37.8	39.2	38.2	38.2

^a^ Six different diets with whole‐crop maize as the main component were prepared from either fresh or ensiled maize. For treatments with fresh maize: fresh wild‐type maize [Fresh wild‐type], fresh bx1 mutant maize [Fresh bx1], fresh bx1 mutant maize plus 250 µg of exogenous MBOA [Fresh bx1+MBOA]. For treatments with ensiled maize: ensiled wild‐type maize [Silage wild‐type], ensiled bx1 mutant maize [Silage bx1] and ensiled bx1 mutant maize plus 250 µg of exogenous MBOA [Silage bx1+MBOA].

^b^ Biomin M16 (Biomin Holding GmbH, Getzersdorf, Austria), composition per kg fresh matter: calcium 19.2%; phosphorus 4%; sodium 11%; magnesium 4%; vitamin A 800,000 IE; vitamin D_3_ 75,000 IE; vitamin E 2000 mg; manganese 4500 mg; iodine 120 mg; zinc 6600 mg; cobalt 90 mg; selenium 40 mg; copper 1000 mg.

^c^ Calculated as dry matter−ash.

^d^ Calculated as 100–ash–crude protein–ether extract–neutral detergent fibre.

### 2.2 Experimental Design and Rusitec Procedure

The present in vitro experiment was performed at the Center for Animal Nutrition and Welfare, University of Veterinary Medicine, Vienna, Austria. The incubations were conducted in the in vitro Rusitec system (Czerkawski and Breckenridge 1977), consisting of 12 fermentation vessels with a capacity of 800 mL, and using a similar setup as explained in detail by Mahmood et al. (2020). Thereby, the six treatments were allocated to 12 vessels in a completely randomized group design during three experimental runs, resulting in six independent replicates per treatment (Robinson et al. 2006; Udén et al. 2012). Each experimental run consisted of 10 days with the first 5 days as an adaptation period and Days 6−10 as a sampling period. During the adaptation period, all vessels were incubated with 12 g dry matter (DM) of the same adaptation diet, which was also based on maize silage (Table 1). From Days 6 to 10, 12 g DM of the respective treatment diet were incubated. The experimental setup and daily management of the Rusitec system was performed as described in detail in Mahmood et al. (2020). In brief, ruminal fluid and solid digesta were obtained from two dry, rumen‐cannulated Holstein cows at about 3 h after the morning feeding on Day 0. The donor cows were fed hay ad libitum plus daily 1 kg of concentrate (KuhKorn PLUS Energie, Garant‐Tiernahrung GmbH, Austria) and kept according to the Austrian guidelines 114 of animal welfare (BGBl. II Nr. 485/2004 idF BGBl. II Nr. 151/2017) at the Clinical Centre for Ruminant and Camelid Medicine, University of Veterinary Medicine Vienna. On Day 0, each vessel was filled with one nylon bag (120 mm × 65 mm, pore size 100 µm) with solid digesta and another nylon bag filled with the adaptation diet. On the following days, only nylon bags with adaptation or treatment diets were placed in the vessels during daily bag exchange. The buffer solution (McDougall 1948) was continuously infused at a rate of 360 mL day^−1^.

### 2.3 Direct Measurements and Sample Collections

The pH and redox potential as well as effluent volumes, were recorded daily to control the correct function of the Rusitec system. Thereby, a measuring device equipped with both a pH electrode and a redox electrode (pH metre Seven Excellence, Mettler‐Toledo GmbH, Switzerland) was used to determine pH and redox potential simultaneously. During the sampling period, the gas production volume of each vessel was determined using the water displacement method and the gas composition was analysed for methane and carbon dioxide using a biogas monitor (Atex Biogas Monitor, Check BM 2000, ANSYCO, Germany). Moreover, the feed bags that had been incubated in the vessels for 48 h were collected and stored at −20°C for later analysis. Similarly, 2 mL of the vessel liquid was sampled before feed bag exchange each day of the sampling period and stored at −20°C until further use.

### 2.4 Analysis of Samples

For the analysis of chemical composition, all diets were dried at 65°C in a forced‐air oven for 48 h, while feed bag residues were lyophilized for 24 h. Subsequently, all samples were ground through a 0.5 mm screen in a ZM 200 ultra‐centrifugal mill (Retsch, Haan, Germany). All analyses were performed according to the official guidelines of the Association of German Agricultural Analytic and Research Institutes (VDLUFA 2012): the DM concentration was determined by oven‐drying the samples at 103°C for at least 4 h (method 3.1), the ash concentration was analysed by combustion in a muffle furnace overnight at 580°C (method 8.1), ether extract was determined using the Soxhlet extraction system (method 5.1.2) and crude protein (CP) using the Kjeldahl method (method 4.1.1). Moreover, the Fibretherm FT12 (Gerhardt GmbH & Co. KG, Königswinter, Germany) was used to determine neutral detergent fibre assayed with a heat‐stable α‐amylase (aNDF, method 6.5.1) and acid detergent fibre (ADF, 6.5.2). Consequently, organic matter (OM) was calculated as DM concentration minus ash concentration. The degradability of DM, OM and proximate nutrients was calculated on a DM basis as the amount incubated with the diet in the feed bag minus the amount present in the feed bag residue.

The analysis of individual SCFA (acetate, propionate, butyrate, isobutyrate, valerate, isovalerate) in the vessel liquid samples was carried out using gas chromatography as described before (Hartinger et al. 2022). Briefly, samples were thawed and centrifuged at 20,000 × *g* at 4°C for 25 min. The supernatant was mixed with 0.2 mL HCl (1.8 mol L^−1^) and 0.2 mL of the internal standard (4‐methylvaleric acid, Sigma Aldrich, USA) and then centrifuged again in the same way. Subsequently, supernatant of samples was injected into the GC device (Shimadzu GC Plus with FID detector, Shimadzu, Kyoto, Japan) that was equipped with a 30 m × 0.53 mm i.d. × 0.53 μm capillary column (Trace TR Wax, Thermo Fisher Scientific, Waltham, MA). The ammonia nitrogen concentration was determined using the Berthelot reaction (Hinds and Lowe 1980).

Moreover, concentrations of BXs and the associated downstream metabolites were analysed daily in the vessel liquid and in the solid feedstuff residue sampled on the first and last day of the sampling period. The vessel liquid samples were allowed to thaw on ice and 20 µL were aliquoted for extraction, while the 20.0 ± 0.5 mg of each solid sample were ground in 1.5 mL Eppendorf tubes at low temperature in the presence of liquid nitrogen. The extraction of BXs was performed by adding 200 µL extraction buffer (composition: HPLC grade methanol, 70% in MilliQ water + 0.1% Optima grade formic acid), vortexing and centrifuging for 20 min at 13,000 rpm and 10°C.

The following compounds were analysed in the supernatants: 2‐β‐d‐glucopyranosyloxy‐4‐hydroxy‐2*H*‐1,4‐benzoxazin‐3(4*H*)‐one (DIBOA‐Glc); 2‐β‐d‐glucopyranosyloxy‐4‐hydroxy‐7‐methoxy‐2*H*‐1,4‐benzoxazin‐3(4*H*)‐one (DIMBOA‐Glc); 2‐β‐d‐glucopyranosyloxy‐4‐hydroxy‐7,8‐dimethoxy‐2*H*‐1,4‐benzoxazin‐3(4*H*)‐one (DIM2BOA‐Glc); 2‐β‐d‐glucopyranosyloxy‐2*H*‐1,4‐benzoxazin‐3(4*H*)‐one (HBOA‐Glc); 2‐β‐d‐glucopyranosyloxy‐7‐methoxy‐2*H*‐1,4‐benzoxazin‐3(4*H*)‐one (HMBOA‐Glc); 2‐β‐d‐glucopyranosyloxy‐4,7‐dimethoxy‐2*H*‐1,4‐benzoxazin‐3(4*H*)‐one (HDMBOA‐Glc); 2‐β‐d‐glucopyranosyloxy‐7,8‐dimethoxy‐2*H*‐1,4‐benzoxazin‐3(4*H*)‐one (HM2BOA‐Glc); 2‐β‐d‐glucopyranosyloxy‐4,7,8‐trimethoxy‐2*H*‐1,4‐benzoxazin‐3(4*H*)‐one (HDM2BOA‐Glc); 3‐β‐d‐glucopyranosyl‐6‐methoxy‐benzoxazolin‐2(3*H*)‐one (MBOA‐Glc); 2,4‐Dihydroxy‐7‐methoxy‐2*H*‐1,4‐benzoxazin‐3(4*H*)‐one (DIMBOA); 2‐Hydroxy‐7‐methoxy‐2*H*‐1,4‐benzoxazin‐3(4*H*)‐one (HMBOA); 6‐Methoxy‐benzoxazolin‐2(3*H*)‐one (MBOA); Benzoxazolin‐2(3*H*)‐one (BOA); 2‐Amino‐3*H*‐phenoxazin‐3‐one (APO); 9‐methoxy‐2‐amino‐3*H*‐phenoxazin‐3‐one (AMPO); 9‐methoxy‐2‐acetylamino‐3*H*‐phenoxazin‐3‐one (AAMPO); *N*‐(3‐methoxy‐2‐hydroxyphenyl)malonamic acid (HMPMA). These compounds were analysed using an Acquity UHPLC system coupled to a G2‐XS QTOF mass spectrometer equipped with an electrospray source (Waters Corporation, USA) as described elsewhere (Gross, Mateo, Ramhold, et al. 2023; Gross, Mateo, Schlaeppi, et al. 2023). Briefly, a gradient elution was performed on an Acquity BEH C18 column (2.1 × 50 mm i.d., 1.7 mm particle size) at 90%−70% A over 3 min, 70%−60% A over 1 min, 40%−100% B over 1 min, holding at 100% B for 2.5 min, holding at 90% A for 1.5 min where A = 0.1% formic acid/water and B = 0.1% formic acid/acetonitrile. The flow rate was 0.4 mL/min. The temperature of the column was maintained at 40°C, and the injection volume was 1 µL. The QTOF MS was operated in positive mode. The data were acquired over an m/z range of 50–1200 with scans of 0.15 s at a collision energy of 4 V and 0.2 s with a collision energy ramp from 10 to 40 V. The capillary and cone voltages were set to 2 kV and 20 V, respectively. The source temperature was maintained at 140°C, the desolvation was 400°C at 1000 L/h and cone gas flow was 50 L/h. Accurate mass measurements (< 2 ppm) were obtained by infusing a solution of leucin encephalin at 200 ng/mL at a flow rate of 10 mL/min through the Lock Spray probe (Waters). The quantification of the compounds was performed using external standard curves (MBOA‐Glc, HMPMA, DIMBOA‐Glc, HMBOA, DIMBOA, BOA, HDMBOA‐Glc, MBOA, APO, AMPO and AAMPO). MBOA and BOA were purchased at Sigma‐Aldrich, Merck & Cie (Shaffhausen, CH). HMPMA was obtained as a gift from Prof. Dr. Francisco A. Macías, Department of Organic Chemistry, Institute of Biomolecules (INBIO), Cadiz, Spain. All the other compounds were obtained through isolation from natural sources or chemical synthesis in our laboratory. When no analytical standard were available, the compounds were quantified on the closest parent.

### 2.5 Statistical Analysis

The statistical analysis was performed using the SAS software version 9.2 (SAS Institute Inc., USA). First, normal distribution was checked using PROC UNIVARIATE. The data were then analysed in a variance analysis according to the completely randomized group design using PROC MIXED, including the fixed effect of the experimental diet, while experimental run were considered as random effects. The compound symmetry was used to account for repeated measurements in the same experimental unit within an experimental run. The degrees of freedom were approximated according to Kenward and Roger (1997). The post hoc multiple mean comparisons were carried out using the Tukey test. Besides, orthogonal contrasts were determined to better discriminate for differences between (i) the impact of conservation of maize, i.e., fresh ([Fresh wild‐type], [Fresh bx1], [Fresh bx1+MBOA]) versus ensiled maize ([Silage wild‐type], [Silage bx1], [Silage bx1+MBOA]), (ii) the impact of genotypes, i.e., wild‐type ([Fresh wild‐type], [Silage wild‐type]) versus bx1 mutant ([Fresh bx1], [Silage bx1]) and (iii) impact of exogenous MBOA addition in the bx1 mutant genotype only, i.e., bx1 ([Fresh bx1], [Silage bx1]) versus bx1+MBOA ([Fresh bx1+MBOA], [Silage bx1+MBOA]). For all variables, significance was defined at *p* ≤ 0.05.

## 3 Results

### 3.1 Concentrations of BXs in the Vessels

As shown in Table 2, three BX‐associated compounds were detected in the solid feedstuff residues of several treatments, namely 6‐Methoxy‐benzoxazolin‐2(3*H*)‐one (MBOA), 2‐Hydroxy‐7‐methoxy‐2*H*‐1,4‐benzoxazin‐3(4*H*)‐one (HMBOA) and 2,4‐Dihydroxy‐7‐methoxy‐2*H*‐1,4‐benzoxazin‐3(4*H*)‐one (DIMBOA). The concentrations of these compounds were not affected by treatment or different during the analysis of orthogonal contrasts (each *p* > 0.05). Regarding the vessel liquid, all BX‐associated compounds remained below the detection limit in all treatments during the complete experiment—only minor traces of MBOA were detected on 1 day in one replicate of [Fresh bx1+MBOA] (14 ng mL^−1^) and two replicates of [Fresh wild‐type] (21 ng mL^−1^), as well as HMBOA was present on 1 day in one replicate of the treatment [Silage wild‐type] (56 ng mL^−1^).

**Table 2:** Concentrations of 6‐methoxy‐2‐benzoxazolinone (MBOA), 2‐hydroxy‐7‐methoxy‐1,4‐benzoxazin‐3‐one (HMBOA) and 2,4‐dihydroxy‐7‐methoxy‐1,4‐benzoxazin‐3‐one (DIMBOA) in the solid feedstuff residue. All values are given in ng g^−^^1^ fresh matter.

								Orthogonal contrasts^b^		
Metabolite^c^	Treatments^a^	Mean	SD	Min	Max	Median	*p* value	Fresh versus silage	Wild‐type versus bx1	bx1 versus bx1+MBOA
HMBOA	Fresh wild‐type	210.9	292.7	0.0	633.5	105.0	0.283	0.932	0.102	0.838
	Fresh bx1	151.4	226.0	0.0	472.5	66.5				
	Fresh bx1+MBOA	35.9	54.3	0.0	108.5	17.5				
	Silage wild‐type	275.6	338.6	0.0	759.5	171.5				
	Silage bx1	42.9	54.0	0.0	115.5	28.0				
	Silage bx1+MBOA	61.3	67.0	21.0	161.0	31.5				
DIMBOA	Fresh wild‐type	20.1	40.3	0.0	80.5	0.0	0.523	0.210	0.484	1.000
	Fresh bx1	53.4	106.8	0.0	213.5	0.0				
	Fresh bx1+MBOA	0.0	0.0	0.0	0.0	0.0				
	Silage wild‐type	0.0	0.0	0.0	0.0	0.0				
	Silage bx1	0.0	0.0	0.0	0.0	0.0				
	Silage bx1+MBOA	0.0	0.0	0.0	0.0	0.0				
MBOA	Fresh wild‐type	113.8	162.8	0.0	353.5	50.8	0.418	0.580	0.658	0.225
	Fresh bx1	193.4	219.9	0.0	479.5	147.0				
	Fresh bx1+MBOA	49.9	70.0	0.0	136.5	31.5				
	Silage wild‐type	210.0	307.3	0.0	665.0	87.5				
	Silage bx1	72.6	72.7	0.0	147.0	71.8				
	Silage bx1+MBOA	162.8	87.1	56.0	252.0	171.5				

^a^ Six different diets with whole‐crop maize as the main component were prepared: (i) fresh wild‐type maize [Fresh wild‐type], (ii) fresh bx1 mutant maize [Fresh bx1], (iii) fresh bx1 mutant maize plus 250 µg of exogenous MBOA [Fresh bx1+MBOA], (iv) Ensiled wild‐type maize [Silage wild‐type], (v) ensiled bx1 mutant maize [Silage bx1] and (vi) ensiled bx1 mutant maize plus 250 µg of exogenous MBOA [Silage bx1+MBOA].

^b^ Orthogonal contrasts determining (i) the impact of conservation of maize, i.e., fresh ([Fresh wild‐type], [Fresh bx1], [Fresh bx1+MBOA]) versus ensiled maize ([Silage wild‐type], [Silage bx1], [Silage bx1+MBOA]), (ii) the impact of genotypes, i.e., wild‐type ([Fresh wild‐type], [Silage wild‐type]) versus bx1 mutant ([Fresh bx1], [Silage bx1]) and (iii) impact of exogenous MBOA addition in the bx1 mutant genotype only, i.e., bx1 ([Fresh bx1], [Silage bx1]) versus bx1+MBOA ([Fresh bx1+MBOA], [Silage bx1+MBOA]).

^c^ The following metabolites were not detected: MBOA‐Glc, HMPMA, DIMBOA‐Glc, BOA HDMBOA‐Glc, APO, AMPO, AAMPO, HBOA‐Glc, DIBOA‐Glc, HMBOA‐Glc, HM2BOA‐Glc, DIM2BOA‐Glc and HDM2BOA‐Glc.

### 3.2 In Vitro Rumen Fermentation Pattern

The analysed in vitro rumen fermentation characteristics are presented in Table 3. The mean pH ranged from 6.49 to 6.54 during the experimental periods and the orthogonal contrast of fresh versus ensiled maize revealed a lower pH during incubations of ensiled maize (*p* = 0.025). The redox potential was affected by the incubated diet (*p* = 0.021) with lower values in [Silage bx1+MBOA] than for [Silage wild‐type] and [Silage bx1], whereas diets with fresh maize did not differ. The concentration of ammonia nitrogen was different between treatments (*p* < 0.001) and higher during incubation of ensiled maize compared to fresh maize, which was also evident from the orthogonal contrast (*p* < 0.001). The concentration of total SCFA ranged between 99.3 and 102.1 mmol L^−1^ but was not different between incubated diets (*p* = 0.795). Regarding individual SCFA, butyrate was higher in [Fresh bx1] than in [Fresh wild‐type], whereas other diets did not differ (*p* = 0.017). The orthogonal contrast analysis showed lower proportions of acetate (*p* = 0.029) and valerate (*p* = 0.013) with ensiled maize compared to fresh maize, whereas propionate proportions were higher with ensiled compared than with fresh maize (*p* = 0.007). In specific, propionate proportions were higher for [Silage bx1] and [Silage bx1+MBOA] than for [Fresh bx1] and [Silage wild‐type] (*p* = 0.006). Likewise, the ratio of acetate to propionate was higher for fresh than for ensiled maize (*p* < 0.001), which, however, was only true for the bx1 mutant genotype (*p* < 0.001). Moreover, the orthogonal contrast of wild‐type versus bx1 mutant revealed higher proportions of isovalerate in the wild‐type genotype (*p* = 0.038), whereas a higher butyrate proportion was present in the bx1 mutant than in the wild‐type genotype (*p* = 0.020).

**Table 3:** Differences in in vitro rumen fermentation pattern between diets.

	Treatments^1^								Orthogonal contrasts^2^		
	Fresh			Silage							
Item	Wild‐type	bx1	bx1+ MBOA	Wild‐type	bx1	bx1+ MBOA	SEM	*p* value	Fresh versus Silage	Wildtype versus bx1	bx1 versus bx1+MBOA
pH	6.52	6.54	6.52	6.50	6.49	6.51	0.05	0.280	0.025	0.991	0.966
Redox (mV)	−240.70^ab^	−237.1^ab^	−226.90^ab^	−223.6^a^	−224.60^a^	−252.50^b^	11.10	0.021	0.794	0.931	0.154
Ammonia nitrogen (mmol L^−1^)	9.46^b^	9.20^b^	9.29^b^	10.31^a^	10.48^a^	10.29^a^	0.43	< 0.001	< 0.001	0.220	0.647
Total SCFA^3^ (mmol L^−1^)	100.40	99.30	101.50	100.00	102.10	100.60	10.60	0.795	0.811	0.818	0.878
Acetate (%^4^)	48.70	48.60	49.10	48.70	48.10	48.00	1.20	0.089	0.029	0.184	0.477
Propionate (%)	23.70^ab^	22.70^b^	23.10^ab^	23.20a^b^	24.10^a^	24.20^a^	1.00	0.006	0.007	0.781	0.328
Butyrate (%)	12.80^b^	14.40^a^	13.20^ab^	13.50^ab^	13.30^ab^	13.90^ab^	0.70	0.017	0.770	0.020	0.285
Isobutyrate (%)	0.97	0.94	0.93	0.95	0.96	0.94	0.03	0.077	0.698	0.143	0.147
Valerate (%)	6.80	6.70	6.80	6.50	6.60	6.40	0.30	0.174	0.013	0.767	0.384
Isovalerate (%)	3.30	3.20	3.30	3.40	3.30	3.20	0.20	0.172	0.713	0.038	0.898
Ratio of acetate to propionate	2.07^ab^	2.18^a^	2.16^a^	2.12^ab^	2.01^b^	2.00^b^	0.13	< 0.001	< 0.001	0.073	0.746

*Note:* Different superscript letters within a row indicate significant difference, i.e., *p* < 0.05, according to a post hoc test.

^1^ Six different diets with whole‐crop maize as the main component were prepared: (i) fresh wild‐type maize [Fresh wild‐type], (ii) fresh bx1 mutant maize [Fresh bx1], (iii) fresh bx1 mutant maize plus 250 µg of exogenous MBOA [Fresh bx1+MBOA], (iv) Ensiled wild‐type maize [Silage wild‐type], (v) ensiled bx1 mutant maize [Silage bx1] and (vi) ensiled bx1 mutant maize plus 250 µg of exogenous MBOA [Silage bx1+MBOA].

^2^ Orthogonal contrasts determining (i) the impact of conservation of maize, i.e., fresh (Fresh wild‐type, Fresh bx1, Fresh bx1+MBOA) versus ensiled maize (Silage wild‐type, Silage bx1, Silage bx1+MBOA), (ii) the impact of genotypes, that is, wild‐type (Fresh wild‐type, Silage wild‐type) versus bx1 mutant (Fresh bx1, Silage bx1) and (iii) impact of exogenous MBOA addition in the bx1 mutant genotype only, i.e., bx1 (Fresh bx1, Silage bx1) versus bx1+MBOA (Fresh bx1+MBOA, Silage bx1+MBOA).

^3^ Short‐chain fatty acids.

^4^ Percentage share of total SCFA.

### 3.3 Profiles of Fermentation Gases

The total daily gas production ranged from 1090 to 1100 mL and was not different between the diets (Table 4). Similarly, no differences in daily production of methane and carbon dioxide were present, which was also true for their proportions of total gas produced.

**Table 4:** Daily production and composition of fermentation gases.

	Treatments^a^										
	Fresh			Silage					Orthogonal contrasts^b^		
Item	Wild‐type	bx1	bx1+ MBOA	Wild‐type	bx1	bx1+ MBOA	SEM	*p* value	Fresh versus Silage	Wildtype versus bx1	bx1 versus bx1+ MBOA
Gas production (mL day^−1^)	1090	1090	1100	1100	1100	1100	100.0	0.984	0.705	0.593	0.797
Methane production (mL day^−1^)	131	130	126	133	133	129	10.5	0.897	0.469	0.979	0.374
Carbon dioxide production (mL day^−1^)	916	916	935	917	951	925	86.0	0.974	0.752	0.581	0.913
Methane (%^c^)	12.5	12.5	12.3	13.1	13.5	12.4	0.63	0.645	0.229	0.758	0.302
Carbon dioxide (%)	83.1	82.8	83.1	82.1	81.9	83.1	1.00	0.836	0.360	0.739	0.369

^a^ Six different diets with whole‐crop maize as the main component were prepared: (i) fresh wild‐type maize [Fresh wild‐type], (ii) fresh bx1 mutant maize [Fresh bx1], (iii) fresh bx1 mutant maize plus 250 µg of exogenous MBOA [Fresh bx1+MBOA], (iv) Ensiled wild‐type maize [Silage wild‐type], (v) ensiled bx1 mutant maize [Silage bx1] and (vi) ensiled bx1 mutant maize plus 250 µg of exogenous MBOA [Silage bx1+MBOA].

^b^ Orthogonal contrasts determining (i) the impact of conservation of maize, i.e., fresh (Fresh wild‐type, Fresh bx1, Fresh bx1+MBOA) versus ensiled maize (Silage wild‐type, Silage bx1, Silage bx1+MBOA), (ii) the impact of genotypes, that is, wild‐type (Fresh wild‐type, Silage wild‐type) versus bx1 mutant (Fresh bx1, Silage bx1) and (iii) impact of exogenous MBOA addition in the bx1 mutant genotype only, i.e., bx1 (Fresh bx1, Silage bx1) versus bx1+MBOA (Fresh bx1+MBOA, Silage bx1+MBOA).

^c^ Percentage share of total gas volume.

### 3.4 Degradability of DM, OM and Proximate Nutrients

**Figure 1:**
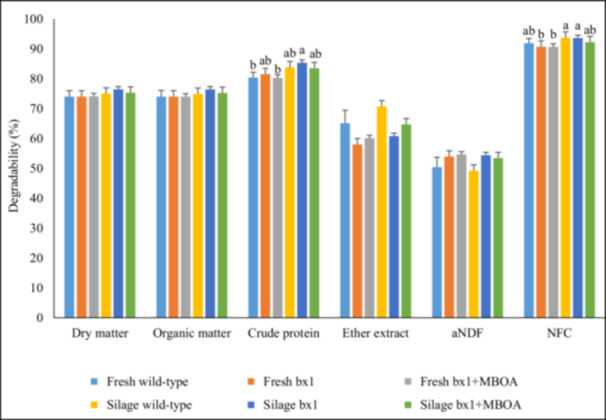
Degradability of dry matter, organic matter, crude protein, ether extract, neutral detergent fibre (aNDF) and non‐fibre carbohydrates (NFC) during 48 h in vitro incubation. Treatments are: (i) fresh wild‐type maize [Fresh wild‐type], (ii) fresh bx1 mutant maize [Fresh bx1], (iii) fresh bx1 mutant maize plus 250 µg of exogenous MBOA [Fresh bx1+MBOA], (iv) Ensiled wild‐type maize [Silage wild‐type], (v) ensiled bx1 mutant maize [Silage bx1] and (vi) ensiled bx1 mutant maize plus 250 µg of exogenous MBOA [Silage bx1+MBOA]. Different superscript letters indicate significant difference, i.e., *p* < 0.05, according to a post hoc test. [Color figure can be viewed at wileyonlinelibrary.com]

The in vitro degradabilities are illustrated in Figure 1. The CP degradability was higher for [Silage bx1] than for [Fresh wild‐type], while the other treatments did not differ (*p* < 0.01). The degradability of NFC was higher for [Silage wild‐type] and [Silage bx1] than for [Fresh bx1] and [Fresh bx1+MBOA] (*p* = 0.02). The analysis of the orthogonal contrasts showed that the incubation of ensiled maize resulted in higher degradability of DM (*p* = 0.028), OM (*p* = 0.027) and NFC (*p* < 0.001) when compared to the incubation of fresh maize. Moreover, aNDF degradability was higher for the bx1 mutant than for the wild‐type maize genotype (*p* = 0.045), whereas ether extract degradability was higher for the wild‐type than for the bx1 mutant maize genotype (*p* = 0.013).

## 4 Discussion

The group of BXs, and in particular the downstream metabolite MBOA, represents secondary plant compounds found in maize with high efficacy against insect pests and plant pathogens (Campos et al. 1988; Schlaeppi et al. 2021). The potential impact of BXs on the ruminal fermentation remains yet unknown, but is indeed worth of investigation since antimicrobial effects may not be limited to insects and plant pathogens but could also harm the microbial activity in the rumen—as at least indicated by research in the rodent gut (Dai et al. 2022). This may be especially a hazard when feeding silage produced from maize genotypes with high native BX concentrations, as the ensiling process enhances the conversion of BXs into MBOA (Gross, Mateo, Schlaeppi, et al. 2023).

The most important finding of the present study was the absence of negative effects on the in vitro fermentation pattern when incubating maize genotypes differing in native BX concentration or when adding exogenous MBOA to the system. Neither pH, proximate nutrient degradabilities nor gas production and composition showed a response to the dietary interventions, which therefore indicated that the microbial community remained stable without interferences in the fermentative activity by BX or exogenous MBOA. Likewise, SCFA profiles were marginally affected and although statistically significant changes for the proportions of butyrate and both branched‐chain SCFA were found between wild‐type and bx1 mutant genotype. These differences may be considered biologically not meaningful, as they remained within typical ranges determined during ruminal fermentation, both in vitro and in vivo, i.e., ~0.7%−0.14%, ~1.0%−5.7% and 8%−13% for isobutyrate, isovalerate and n‐butyrate, respectively (Khiaosa‐ard et al. 2015; Hartinger et al. 2019; Deitmers et al. 2024). Consequently, the hypothesis of a detrimental effect of BXs on the in vitro ruminal fermentation was refuted, irrespectively whether BXs derived from plant‐native or exogenous origin. Similar to the majority of other fermentation characteristics, there were no differences observed in ammonia nitrogen concentrations between the treatments. Therefore, the inhibition of proteolytic microorganisms and their activity in the rumen, which is described for a variety of secondary plant compounds (Hartinger et al. 2018), can be ruled out for BXs, too. This implies that there are no improvements in nitrogen utilization in ruminants associated with BXs. Since BXs were generally below the detection limit in the vessel liquid, it can be assumed that the degradation capacity of the rumen microbiome is strong enough to sufficiently neutralize BXs in a rapid manner. It can be speculated whether the microorganisms utilized MBOA as a carbon source for biomass growth as already observed in maize root bacteria (Thoenen et al. 2024)—especially since no typical MBOA degradation products were detected in the vessel liquid, including HMBOA and DIMBOA as well AMPO that is a main MBOA degradation product in the rhizosphere of maize (Macías et al. 2004; Thoenen et al. 2024). Moreover, only three BXs were found in low concentrations in the solid feedstuff residue of a few treatments, which may be ascribed to BXs trapped in feed particles and so not accessible for degradation by the rumen microbes.

Notably, differences in in vitro fermentation between incubations of fresh versus ensiled maize were observed in the present study. Compared to fresh maize, ensiled maize resulted in an increased degradability of DM, OM, CP and NFC with concomitantly elevated levels of propionate and ammonia nitrogen in the vessels. These effects on substrate degradability and fermentation pattern may likely be not associated with differences in MBOA loads, but rather the impact of the ensiling process on protein‐starch‐matrices that solubilize during silo storage and thereby facilitate microbial attachment and degradation of starch and CP in the rumen (Hoffman et al. 2011; Cueva et al. 2023). Likewise, the simultaneously slightly lower acetate proportions indicated a reduced fibre fermentation in ensiled versus fresh maize, presumably since easily fermentable hemicelluloses are already mostly degraded in the silo via acid hydrolysis (Dewar et al. 1963). In addition, this assumption of the ensiling process and not BXs being causative was also supported by the lacking effect of exogenous MBOA application on degradability of DM, OM and proximate nutrients.

Unambiguously, the present data provide the first evidence about the non‐harmful character of BXs in the rumen ecosystem. Despite the similar fermentative activity of the microbes across all treatments, shifts in the microbial composition and structure may have occurred but remained undetected due to the functional redundancy in the rumen microbiome (Firkins and Yu 2015). As this cannot be resolved with the present data, future studies are needed addressing this aspect.

Nonetheless, for a more holistic risk assessment, acknowledging the one health concept, it is essential to consider the post‐rumen fate of this secondary plant compound. Prior studies with other mammals, such as rats, pigs and humans, have consistently demonstrated that a considerable proportion of ingested BXs is not excreted via urine or faeces, suggesting intestinal absorption (Adhikari et al. 2015). This may also apply to ruminants, where this possibility remains yet unexplored. Indeed, no BX were detected in the vessel liquid, and so it is conceivable that at least a part of these secondary compounds was not microbially degraded but washed out from the vessels, meaning they could enter the lower gut in the animal. Likewise, low concentrations of MBOA, HMBOA and DIMBOA were detected in the solid feedstuff residue that may then also enter the bovine small intestine. Moreover, although the rapid BX neutralization in the vessels can be fully ascribed to the rumen microbes since the Rusitec system comprises no absorptive site, there may be a potential BX absorption in the forestomach system under in vivo conditions. In addition, the ruminal BX neutralization capacity may not be a fixed figure but also depend on feeding regime as also seen for mycotoxin degradation that is lower in high‐grain fed compared to forage‐fed cattle (Seeling and Dänicke 2005). Therefore, since the present study found no detrimental impact of BXs on the ruminal fermentation, these findings may build the basis for future in vivo trials investigating the absorption and metabolism of BXs in ruminants. Additionally, future studies should explore the potential carry‐over of these plant secondary compounds into milk and meat.

## 5 Conclusion

The present study found no negative impact on the long‐term in vitro ruminal fermentation and proximate nutrient degradation when incubating a low and a high BX maize genotype, either in fresh or ensiled form as part of a dairy diet in the Rusitec system. Future studies may verify the present findings in vivo and determine the potential absorption and metabolization by the animal.

## Ethics Statement

The authors confirm that the ethical policies of the journal, as noted on the journal's author guidelines page, have been adhered to. No ethical approval was required.

## Conflicts of Interest

The authors declare no conflicts of interest.

## Data Availability

The data that support the findings of this study are available from the corresponding author upon reasonable request.
